# Pre-treatment with IL-6 potentiates β-cell death induced by pro-inflammatory cytokines

**DOI:** 10.1186/s12860-023-00476-3

**Published:** 2023-03-28

**Authors:** V. R. Oliveira, C. C. Paula, S. Taniguchi, F. Ortis

**Affiliations:** Department of Cell and Developmental Biology, Institute of Biomedical Sciences, University of São Paulo, São Paulo, Brazil

**Keywords:** β-cells, NF-κB, Pro-inflammatory cytokines, IL-6, ER stress and TUDCA

## Abstract

**Background:**

Type I Diabetes *mellitus* (T1D) is characterized by a specific destruction of β-cells by the immune system. During this process pro-inflammatory cytokines are released in the pancreatic islets and contribute for β-cells demise. Cytokine-induced iNOS activation, via NF-κB, is implicated in induction of β-cells death, which includes ER stress activation. Physical exercise has been used as an adjunct for better glycemic control in patients with T1D, since it is able to increase glucose uptake independent of insulin. Recently, it was observed that the release of IL-6 by skeletal muscle, during physical exercise, could prevent β-cells death induced by pro-inflammatory cytokines. However, the molecular mechanisms involved in this beneficial effect on β-cells are not yet completely elucidated. Our aim was to evaluate the effect of IL-6 on β-cells exposed to pro-inflammatory cytokines.

**Results:**

Pre-treatment with IL-6 sensitized INS-1E cells to cytokine-induced cell death, increasing cytokine-induced iNOS and Caspase-3 expression. Under these conditions, however, there was a decrease in cytokines-induced p-eIF2-α but not p-IRE1expression, proteins related to ER stress. To address if this prevention of adequate UPR response is involved in the increase in β-cells death markers induced by IL-6 pre-treatment, we used a chemical chaperone (TUDCA), which improves ER folding capacity. Use of TUDCA increased cytokines-induced Caspase-3 expression and Bax/Bcl-2 ratio in the presence of IL-6 pre-treatment. However, there is no modulation of p-eIF2-α expression by TUDCA in this condition, with increase of CHOP expression.

**Conclusion:**

Treatment with IL-6 alone is not beneficial for β-cells, leading to increased cell death markers and impaired UPR activation. In addition, TUDCA has not been able to restore ER homeostasis or improve β-cells viability under this condition, suggesting that other mechanisms may be involved.

**Supplementary Information:**

The online version contains supplementary material available at 10.1186/s12860-023-00476-3.

## Background

Type I Diabetes *mellitus* (T1D) is an autoimmune disease characterized by a genetic predisposition associated with environmental factors, that results in an autoimmune attack against insulin-producing β-cells, leading to its dysfunction and death [1–6].

This specific attack against β-cells occurs through a process known as insulitis, in which cells of the immune system infiltrate pancreatic islets and secret pro-inflammatory cytokines, such as Interleukin (IL)-1β, Interferon (IFN)-γ and Tumoral Necrosis Factor (TNF) [3, 7]. Leading, among others, to the expression of Inducible Nitric Oxide Synthase (iNOS), with consequently production of nitric oxide (NO) and of Endoplasmic Reticulum (ER) stress induction, triggering β-cells death [1–3, 8–11]. The activation of ER stress induces a cell survival response known as Unfolded Protein Response (UPR), which has the function of recovering homeostasis of ER. However, if the ER homeostasis is not recovered, pro-apoptotic pathways are activated [9, 12–14], as we observe for cytokine-exposed β-cells [9, 13–16].

T1D patients treatment consists of administration of exogenous insulin combined with the practice of physical exercise, which helps to restore glycemic control [17–19] and increase glucose uptake independent of insulin [20–22]. Beside this effect, it has being reported that physical exercise has an anti-inflammatory effect, leading to improvement in insulin secretion and β-cells protection [23–25]. However, the mechanism and pathways involved in this protection are not yet well defined. In the last years, attention has been focused on Interleukin 6 (IL-6) [26, 27], which when is released by skeletal muscles, it can stimulate the release of anti-inflammatory factors such as Interleukin 10 (IL-10) and inhibit the production of pro-inflammatory cytokines such as TNF [20, 28–30].

The protective anti-inflammatory effect of IL-6 in pancreatic β-cells was shown by Paula and collaborators [24, 25], in which islets of human and trained animals were resistant to cell death induced by pro-inflammatory cytokines, with a decrease in Caspase-3 and iNOS expression. Moreover, serum from physical trained subjects protected human and rodent β-cells against ER stress and apoptosis [25]. In addition, blocking of IL-6 partially prevented these beneficial effects [24, 25].

Since IL-6 is a pleiotropic cytokine, that is described to have pro- and anti-inflammatory effects [31], it is important to understand the specific pathways that could be modulated by IL-6 in β-cells, to prevent the deleterious effect of pro-inflammatory cytokines. This knowledge may help to design therapeutic protocols for T1D treatment or even prevention. Thus, here we pre-exposed the rat β-cell line, INS-1E, to IL-6 and then treated these cells with a combination of pro-inflammatory cytokines, to mimetic the insulites environment. We then investigate the effects of this pre-treatment on the expression of pro-apoptotic markers involved in cytokine induced β-cells death.

## Results

### Pre-treatment with IL-6 increases cytokine-induced pancreatic β-cells death markers

**Fig. 1:**
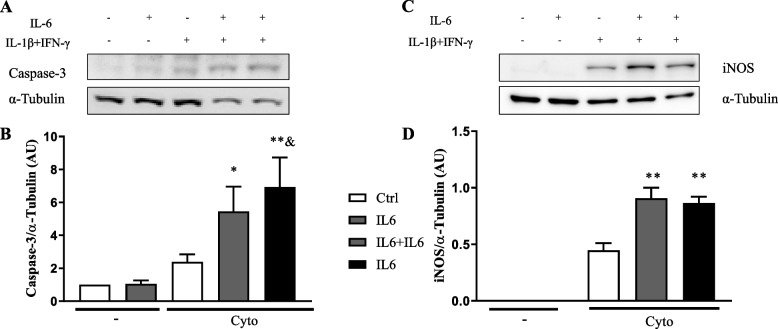
IL-6 increases IL-1β and IFN-γ induced Caspase-3 and iNOS expression in INS-1E cells. Cells were exposed to IL-6 (IL6) or untreated (Ctrl). After 24 h cells were exposed to IL-1β and IFN-γ in the absence (Cyto) or in the presence of pre-treatment with IL-6 (IL6 + Cyto) or continuous treatment of IL-6 (IL6 + IL6 + Cyto). **A** Western blot for Caspase-3 and α-Tubulin. **B** The barplot showing the means values obtained in six independent experiments corrected by the housekeeping protein α-Tubulin. One-way ANOVA followed by Sidak’s correction. *p < 0,05, **p < 0,001 vs Ctrl, &*p* < 0,05 vs Cyto. **C** Western blot for iNOS and α-Tubulin. **D** The barplot showing the mean values obtained in four independent experiments corrected by the housekeeping protein GAPDH. One-way ANOVA followed by Sidak’s correction. **p < 0,001 vs Cyto

Exposure of INS-1E cells to pro-inflammatory cytokines (IL-1β + IFN-γ) leads to cell death, mainly by apoptosis, with increase of Caspase-3 (the active form) and iNOS expression [3, 11], as observed here (Fig. 1).

Pre-exposure of these cells to IL-6 increased cytokines-induced Caspase-3 expression (Fig. 1 A-B), although IL-6 alone had no effect on Caspase-3 expression. Similarly, induction of iNOS expression by cytokines was increased by IL-6 pre-exposure, with no effect of IL-6 alone on iNOS expression (Fig. 1 C-D). Of note, the same effect was observed when IL-6 was only used during the pre-exposure time, 24 h before cytokines exposure, or was kept also after this period together with the cytokine mix.

### Pre-treatment with IL-6 protect β-cells against cytokine-induced UPR protein expression

Cytokine-induced iNOS in rat β-cells leds to NO production and ER stress activation, which contribute to β-cells death [3, 13, 16]. Thus, we evaluate expression of phosphorylated form of eIF2-α (p-eIF2-α) and IRE1 (p-IRE1), two ER stress markers induced by cytokines in β-cells [13, 14, 16].

**Fig. 2:**
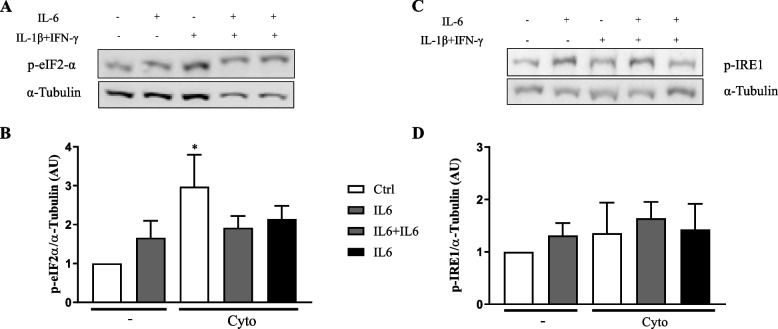
IL-6 decreases IL-1β and IFN-γ induced p-eIF2-α and p-IRE1 expression in INS-1E cells. Cells were exposed to IL-6 (IL6) or untreated (Ctrl). After 24 h cells were exposed to IL-1β and IFN-γ in the absence (Cyto) or in the presence of pre-treatment with IL-6 (IL6 + Cyto) or continuous treatment of IL-6 (IL6 + IL6 + Cyto). **A** Western blot for p-eIF2-α and α-Tubulin (note that α-Tubulin image is the same used in Fig. 1A, since they are from the same blot where caspase 3 and eiF2α were evaluated). **B** The barplot showing the mean values obtained in five independent experiments corrected by the housekeeping protein α-Tubulin. One-way ANOVA followed by Sidak’s correction. *p < 0,05 vs Ctrl. **C** Western blot for p-IRE1 and α-Tubulin. **D** The barplot showing the mean values obtained in four independent experiments corrected by the housekeeping protein α-Tubulin

As expected, exposure of β-cells to cytokines increase expression of p-eIF2-α and p-IRE1 (Fig. 2 A-B and C-D, respectively). Although IL-6 alone did not modulate expression of this ER stress marker, it prevented induction of p-eIF2-α by cytokines (Fig. 2).

### The deleterious effect of IL-6 on pancreatic β cells is not prevented by increasing of ER protein folding capacity

We next used a chemical chaperon, TUDCA, that was previously shown to improve ER protein folding and rescue cells from ER stress [14, 32, 33], to evaluate if this would help to protect β-cells against IL-6 pre-treatment leading to cytokine-induced cell death worsening.

**Fig. 3:**
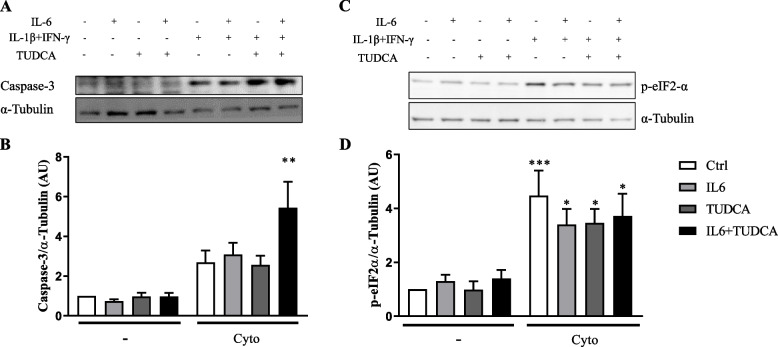
TUDCA increases Caspase-3 but decreases p-eIF2-α expression in INS-1E cells. Cells were exposed to IL-6 (IL6) or untreated (Ctrl). After 24 h cells were exposed to TUDCA in the absence (TUDCA) or in the presence of pre-treatment with IL-6 (IL6 + TUDCA) and IL-1β and IFN-γ (TUDCA + Cyto) or pre-treatment with IL-6 and IL-1β and IFN-γ (IL6 + TUDCA + Cyto). Cells also were exposed to IL-1β and IFN-γ in the absence (Cyto) or in the presence of pre-treatment with IL-6 (IL6 + Cyto). **A** Western blot for Caspase-3 and α-Tubulin. **B** The barplot showing the mean values obtained in six independent experiments corrected by the housekeeping protein α-Tubulin. One-way ANOVA followed by Sidak’s correction. **p < 0,001 vs Ctrl. **C** Western blot for p-eIF2-α and α-Tubulin. **D** The barplot showing the mean values obtained in five independent experiments corrected by the housekeeping protein α-Tubulin. One-way ANOVA followed by Sidak’s correction. *p < 0,05, ***p < 0,0001 vs Ctrl

**Fig. 4:**
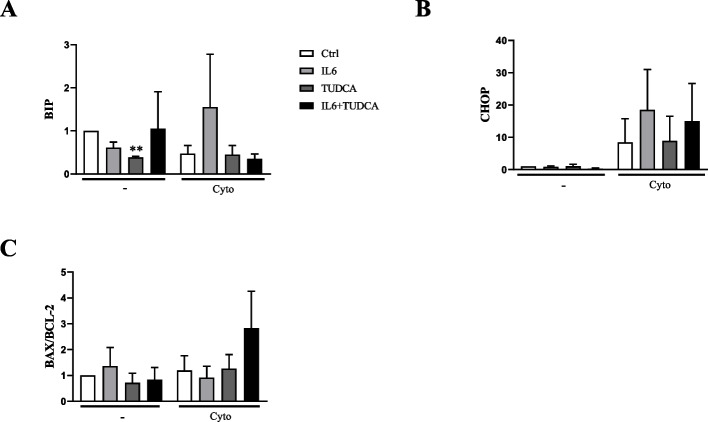
TUDCA prevent increase in BiP expression induced by cytokines + IL6 but not CHOP expression and Bax/Bcl2 ratio in INS-1E cells. Cells were exposed to IL-6 (IL6) or untreated (Ctrl). After 24 h cells were exposed to TUDCA in the absence (TUDCA) or in the presence of pre-treatment with IL-6 (IL6 + TUDCA). Cells also were exposed to IL-1β and IFN-γ (Cyto) or not (-). Expression of BiP **A** and CHOP **B** and Bax/Bcl-2 **C** was evaluated by RT-qPCR. The bar plot shows the mean values obtained in three independent experiments normalized by Rn18s and control by method 2-ΔΔCT. One-way ANOVA followed by Sidak’s correction. **p < 0,001 vs Ctrl

Our results showed that TUDCA addition to condition of pre-treatment with IL-6 and exposure to cytokines seems to further increase Caspase-3 expression (Fig. 3 A-B), in spite of not being able to modulate the expression of p-eIF2-α (Fig. 3 C-D) or BiP mRNA (Fig. 4A) in this conditions. In addition, TUDCA did not prevent increased mRNA expression of CHOP (Fig. 4B) neither of Bax/Bcl-2 ratio (Fig. 4C).

## Discussion

Physical exercise is an important tool in the treatment of numerous diseases, including T1D. Particularly, in the T1D the exercises have been argued as a beneficial factor, closely associated with the increase of β-cells viability and its physiological functions [23–25]. It is known that during physical exercise myokines are produced by skeletal muscle that can result in beneficial effects, among them, IL-6 has been shown to stimulate anti-inflammatory factors release and inhibit of pro-inflammatory cytokines production [20, 28–30]. However, most of these findings are based on the use of serum from exercised animals, which can induce the activation of several pathways in addition to IL-6, leading to a combined beneficial effect to the β-cells in the pro-inflammatory insult that occurs during T1D [23–25]. Therefore, it is important to evaluate the effects of IL-6 alone in pancreatic β-cells exposed to pro-inflammatory cytokines.

It is known that the cytokine-induced nitric oxide production, through NF-κB induced iNOS, is associated with the blocking of the ER calcium pump (SERCA2b) [16, 34] and causes stress in the ER, contributing to β-cells death [3, 13, 16]. Our data showed that pre-treatment with IL-6 leads to a higher sensitivity of INS-1E cells to death induced by pro-inflammatory cytokines, inducing increased iNOS and Caspase-3 expression. These data show us that pre-treatment with IL-6 alone does not have a beneficial effect on INS-1E cells viability exposed to pro-inflammatory cytokines. This apparently contradiction of our results and the beneficial effects of pre-treatment with IL-6 observed by Paula [24] may be due to the different cytokine combination used here. Paula used a combination of IL-1β and IFNγ, here added also TNF, since these three are major cytokines players during insulites [3]. This may indicate that TNF may induce other deleterious pathways that would lead to a pro-apoptotic effect of IL-6 in these cells.

It has recently been shown serum from exercise human or mouse prevent the death of human and rat β-cells exposed to tapsigargin or CPA (inhibitors of the SERCA2b pump), showing an effect of myocins in the ER stress pathways [25]. Our data shows that the pre-treatment of INS-1E cells with IL-6 alone decreases IL-1β and IFN-γ-induced expression of p-eIF2-α and p-IRE1, two important UPR markers, which are induced during ER stress provoked in β-cells exposed to cytokines [14, 16, 35].

Although pre-treatment with IL-6 decrease partially UPR response it still increases expression of Caspase-3 and iNOS expression, indicating increase of β-cells death in this condition. One possible hypothesis is that IL-6 could be decreasing the β-cells ability to activate UPR pathways, losing the ability to improve ER homeostasis, precipitating β-cells demise. It is known that although the activation of UPR in response to IL-1β and IFN-γ contributes to the death of β-cells [3, 9, 13, 14, 16], these pathways are primarily cellular responses to recover function and maintain cell viability [12, 13]. Thus, ER stress is characterized by a cellular response to the accumulation of poorly folded proteins within this organelle, however, it can lead to cell death if fails to restore homeostasis in the ER [12]. Of note, exposure of β-cells to cytokines induces a deficient UPR, which leads to activation of cell death pathways in this cells [7, 11–14, 16, 35, 36].

To solve the problem of poorly folded proteins, one of UPR mechanisms is to increase the expression of chaperones, that bind to unfolded proteins preventing them from aggregating [33, 37, 38]. The use of chemical chaperones to restore ER homeostasis have being reported, with beneficial results in protecting β-cells from ER-stress induced conditions [14, 32, 39]. Administration of TUDCA, a chemical chaperone, has been shown to reduce apoptosis of β-cells in a TD1 mice model and restore ER homeostasis [24], in addition, this protection has also been shown in human β-cells against cytokine-induced apoptosis [14].

Thus, we used TUDCA to assess whether an increase in the folding capacity of poorly folded proteins in the ER could improve the viability of INS-1E cells exposed to IL-6 and IL-1β + IFN-γ. The treatment with TUDCA did not prevent β-cells death under these conditions, indicating that perhaps other pathways may be involved in this process. Furthermore, there was an increase in Bax/Bcl2 mRNA ratio under these conditions, corroborating with lack of cell death prevention. We also observed that under control conditions, administration of TUDCA decrease expression of BiP (Fig. 4A), which could be due to TUDCA working as a chemical chaperone, decreasing the need for BiP expression. However, since BiP is an important chaperone for maintaining ER homeostasis and its decrease can lead to ER stress activation [13, 14], this could contribute for the deleterious effects when these cells are exposed to cytokines in combination with IL-6, since this treatment also lead to impaired ER stress responses, preventing activation of p-eIF2α.

Taking together, this data may indicate that the positive effects by IL-6 on β-cells, observed by other authors, may involve negative modulation of the ER stress, which, together with the activation of additional pathways, induced by other regulators, such as other myocines present in the serum of trained animals, may prevent β-cells death.

## Conclusions

Our data indicate that IL-6 pre-treatment sensitizes INS-1E cells death induced by pro-inflammatory cytokines IL-1β and IFN-γ. Increased iNOS expression in this condition, indicates involvement of NF-κB activation. This pre-treatment, however, prevented cytokine-induced important UPR pathways, namely p-eIF2-α, which may negatively interfere in ER folding capacity and cell viability. Use of the chemical chaperone TUDCA showed that the deleterious effect of pre-treatment with IL-6 may not seem to be related only to a worsening of the protein folding capacity in the ER, but to other mechanisms not yet demonstrated in the literature.

## Methods

### Cell culture

INS-1E cell line, an efficient model to study β-cells, because of their similarity to primary mouse β-cells in both gene expression and insulin secretion in the glucose response [40, 41], was used in this work. This rat insulinoma cells, kindly provided by Prof. Dr. Claes B. Wolheim and Prof. Dr. Pierre Maechler (University of Geneva, Switzerland), was grown in RPMI 1640 culture medium (Gibco/Invitrogen, Carlsbad, CA, USA) supplemented with 5% fetal bovine serum (FBS), 10 mM HEPES, 100U/mL penicillin, 100 µg/mL streptomycin, 1 mM sodium pyruvate and 50 µM 2-mercaptoethanol. The cultures of these cells were kept in an incubator (Autoflow IR Water-Jacketed CO_2_ Incubator, NuAire) at 37ºC, in a humidified atmosphere at 5% CO_2_.

### Cell treatment

The cells were plated (100.000 cells/ condition in 24 wells plate) and cultured for 48 h before any treatment, to guarantee their adherence to the plaque and reduce the effects of stress due to trypsinization. After 48 h, cells were exposed to IL-6 at a concentration of 100 ng/mL (Life Technologies, Grand Island, NY, USA) for 24 h and then exposed to cytokine mix of IL-1β (Promega) at a concentration of 10U/mL and IFN-γ (Promega) at a concentration of 14U/mL, for additional 24 h. This additional exposure was performed in the presence or absence of IL-6 and/or TUDCA (Calbiochem), at a concentration of 300 µM. Unexposed cells were used as a control.

### Western blot

Total protein extracts were fractionated on sodium dodecyl sulphate–polyacrylamide gel electrophoresis (SDS-PAGE) gel at concentrations of 8 or 14%. The proteins were transferred by electrophoresis to nitrocellulose membranes and the detection of specific bands was done using specific primary antibodies for the iNOS protein (#IO117), Caspase-3 (#9662, Cell Signaling), p-eIF-2α (#9721, Cell Signaling), p-IRE1 (#MABC742, Millipore), and as loading control α-Tubulin (#9026, Sigma-Aldrich) and GAPDH (#25,778, Santa Cruz Biotechnology). The membranes were incubated with secondary antibodies (horseradish peroxidase-labeled anti-IgG) anti-mouse IgG (#170–5046, Bio-Rad) or anti-rabbit IgG (#170–5047, Bio-Rad). The chemiluminescence (Ameersham ECL Plus Western Blotting Detection Reagents (GE Healthcare Life Sciences) and the detection of specific bands were made using the image documenter ImageQuant™ LAS 4000 (GE Healthcare Life Sciences). Quantification was performed by densitometry using the ImageQuant™ TL 8.1 (GE Healthcare) and Image Lab 6.1 (Bio-Rad) program and the values were normalized by the internal control densitometry values (α-Tubulin and GAPDH).

### Two-step RT-qPCR

Total RNA was isolated with TRIzol™ Reagent according to the manufacturer’s protocol. Two μg of DNase-treated total RNA (A260/280 ≥ 1,8), was used for cDNA synthesis, performed with SuperScript™ IV according to the manufacturer’s protocol. Quantitative PCR was performed in triplicates with 10 ng of cDNA and 300 nM of specific primers for the reference gene rat (Rn)18 s (F: 5’-TTCCCAGTAAGTGCGGGTCAT-3’ and R: 5’-AGTCAAGTTCGACCGTCTTCTCA-3’), CHOP (F: 5’-CCACACCTGAAAGCAGAAACC-3’ and R: 5’- GCTAGGGATGCAGGGTCAAG-3’), BiP (F: 5’-GCTAGGGATGCAGGGTCAAG-3’ and R: 5’- AAGGGTCATTCCAAGTGCGT-3’), Bax (F: 5’-AGCTGCAGAGGATGATTGCT-3’ and R: 5’-AGCCACCCTGGTCTTGGAT-3’) and Bcl2 (F: 5’- GGCCTTCTTTGAGTTCGGTG-3’ and R: 5’- ATATAGTTCCACAAAGGCATCCCAG) with PowerUp™ SYBR™ Green according to the manufacturer’s protocol in the Rotor-Gene Q. Data was analyzed with the 2-ΔΔCT method, as described previously [42].

### Statistical analysis

The results are presented as means ± SEM of the indicated number of independent experiments and were analyzed statistically, using GraphPad Prism 7 program (La Jolla, CA, USA), by ANOVA followed by Sidak’s correction, as indicated. A < 0.05 *p* value was considered statistically significant.

## Supplementary Information

**Additional file 1.** 

**Additional file 2.**

## Data Availability

All data generated or analysed during this study are included in this published article [and its supplementary information files].

## Abbreviations

T1D: Type one Diabetes
IL-1β: Interleukin-1β
IFN-γ: Interferon-γ
TNF: Tumoral necrosis factor
NF-κB: Nuclear factor kappa B
iNOS: Inducible nitric oxide synthase
NO: Nitric oxide
ER: Endoplasmic reticulum
UPR: Unfolded protein response
TUDCA: Tauroursodeoxycholic
IL-6: Interleukin 6
IL-10: Interleukin 10
p-eIF2-α: Phospho-Eukaryotic initiation factor 2-α
p-IRE1: Phospho-Inositol-requiring enzyme 1
FBS: Fetal bovine serum
SDS-PAGE: Sodium dodecyl sulphate–polyacrylamide gel electrophoresis
IgG: Immunoglobulin G
SERCA2b: Sarcoplasmic/endoplasmic reticulum calcium ATPase 2b
CPA: Cyclopiazonic acid
RT-qPCR: Reverse transcriptase quantitative polymerase chain reaction
