# Supplementary material for: Pre-treatment with IL-6 potentiates β-cell death induced by pro-inflammatory cytokines
Source: BMC Mol Cell Biol. 2023 Mar 28;24:11. doi: 10.1186/s12860-023-00476-3 (PMC10045109; doi:10.1186/s12860-023-00476-3)
Supplement: Supplementary file 2 — Additional file 2. [file 12860_2023_476_MOESM2_ESM.pdf]

Figure 1 A-B. Caspase-3/α-tubulin

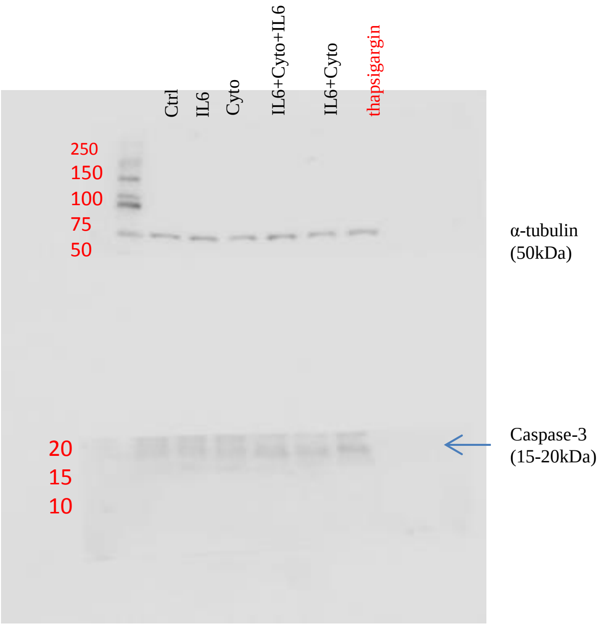

1st experiment

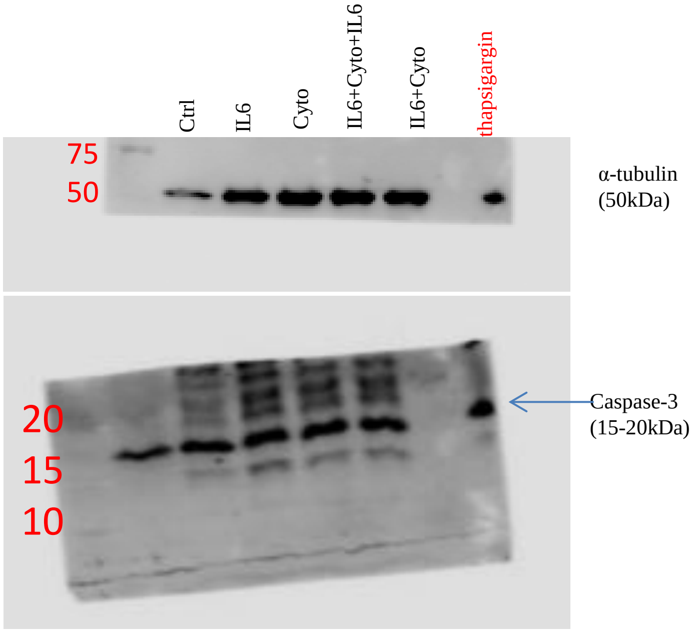

2nd experiment

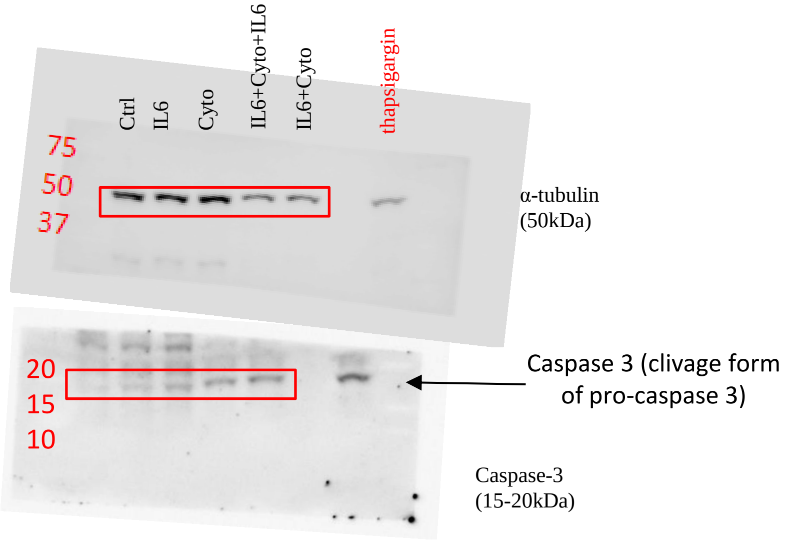

3rd experiment

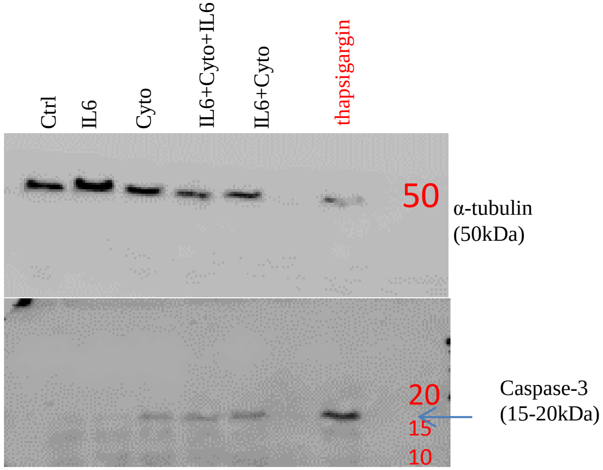

4th experiment

Figure 1 A-B. Caspase-3/α-tubulin

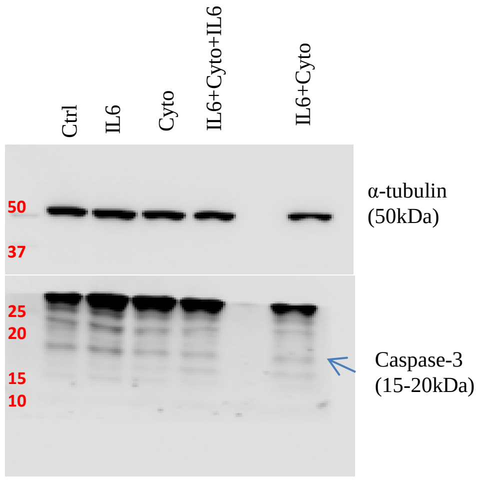

5th experiment

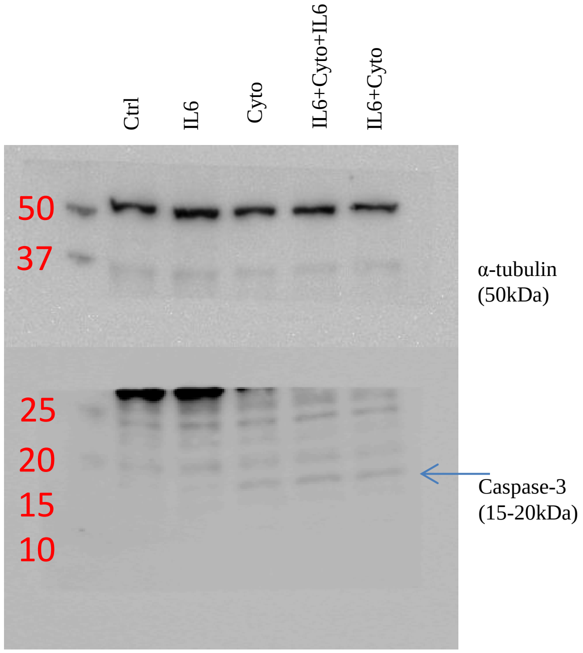

6th experiment

Figure 1 supplementary: Blots for Caspase 3 and tubulin in INS-1E cells. All six independent experiments performed for evaluation of Caspase-3 expression (showed bellow for each experiment) showed in Figure 1 A-B (manuscript) and the correspondent a-tubulin (showed above in each experiment). The red rectangle evidentiate the part of the blot used in figure 1 (manuscript). Cells were exposed to IL-6 (IL6) or untreated (Ctrl). After 24h cells were exposed to IL-1β and IFN-γ in the absence (Cyto) or in the presence of pre-treatment with IL-6 (IL6+Cyto) or continuous treatment of IL-6 (IL6+IL6+Cyto).

Figure 1 C-D. iNOS/α-tubulin

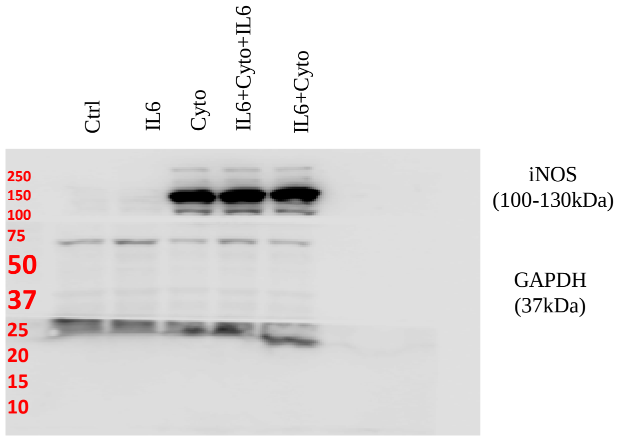

1st experiment

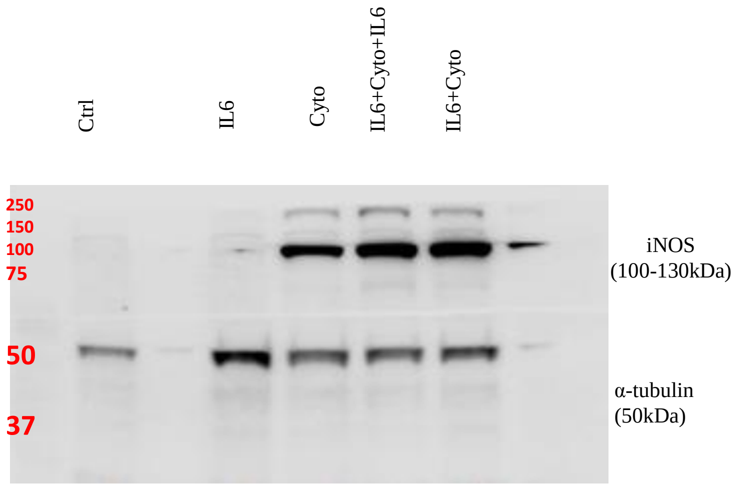

2nd experiment

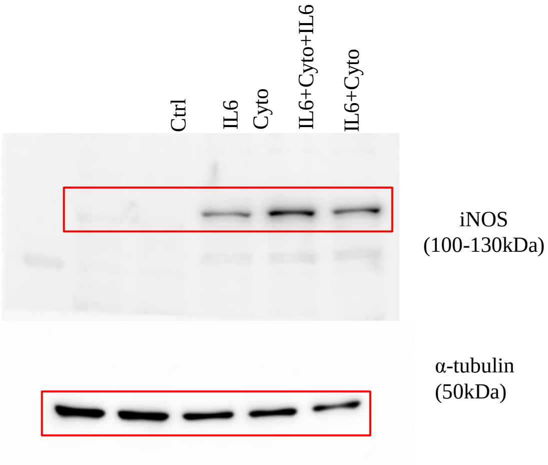

3rd experiment

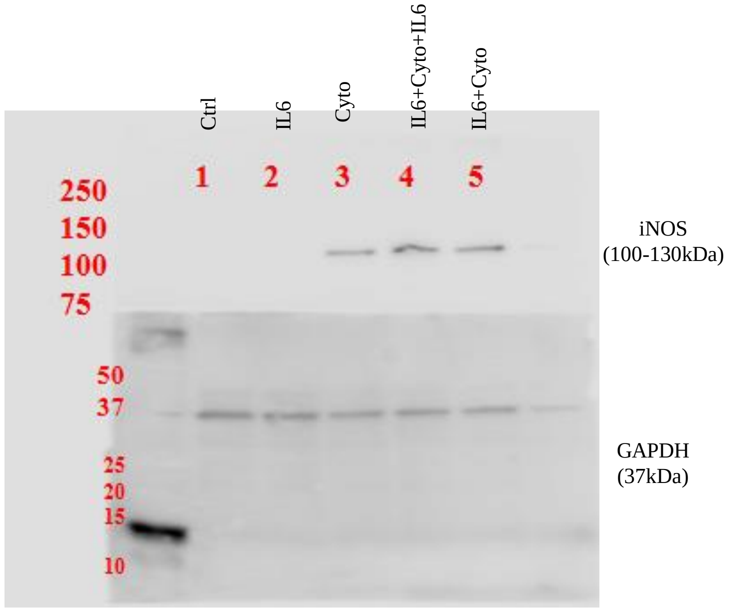

4th experiment

Figure 2 supplementary: Blots for iNOS and GAPDH/α-tubulin in INS-1E cells. All 4 independent experiments performed for evaluation of iNOS expression (showed above for each experiment) showed in Figure 1 C-D (manuscript) and the correspondent GAPDH/α-tubulin (showed bellow in each experiment). The red rectangle evidentiate the part of the blot used in figure 1 (manuscript). Cells were exposed to IL-6 (IL6) or untreated (Ctrl). After 24h cells were exposed to IL-1β and IFN-γ in the absence (Cyto) or in the presence of pre-treatment with IL-6 (IL6+Cyto) or continuous treatment of IL-6 (IL6+IL6+Cyto).

Figure 2 A-B. p-eIF2α/α-tubulin

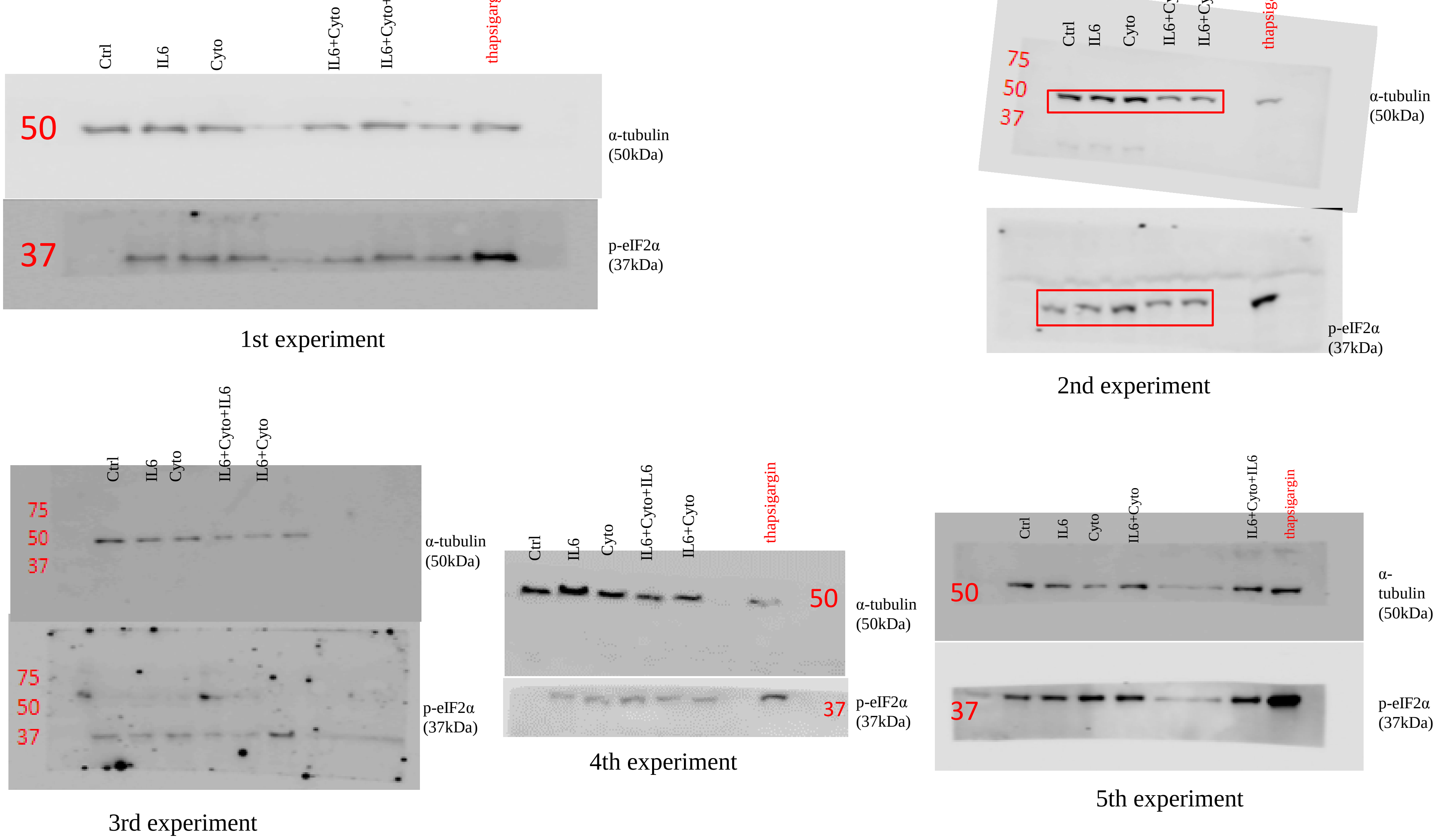

Figure 3 supplementary: Blots for eIF2a and a-tubulin in INS-1E cells. All 5 independent experiments performed for evaluation of eIF2a expression (showed bellow for each experiment) showed in Figure 2 A-B (manuscript) and the correspondent a-tubulin (showed above in each experiment). The red rectangle evidentiate the part of the blot used in figure 2 (manuscript). Cells were exposed to IL-6 (IL6) or untreated (Ctrl). After 24h cells were exposed to IL-1β and IFN-γ in the absence (Cyto) or in the presence of pre-treatment with IL-6 (IL6+Cyto) or continuous treatment of IL-6 (IL6+IL6+Cyto).

Figure 2 C-D. p-IRE1α/α-tubulin

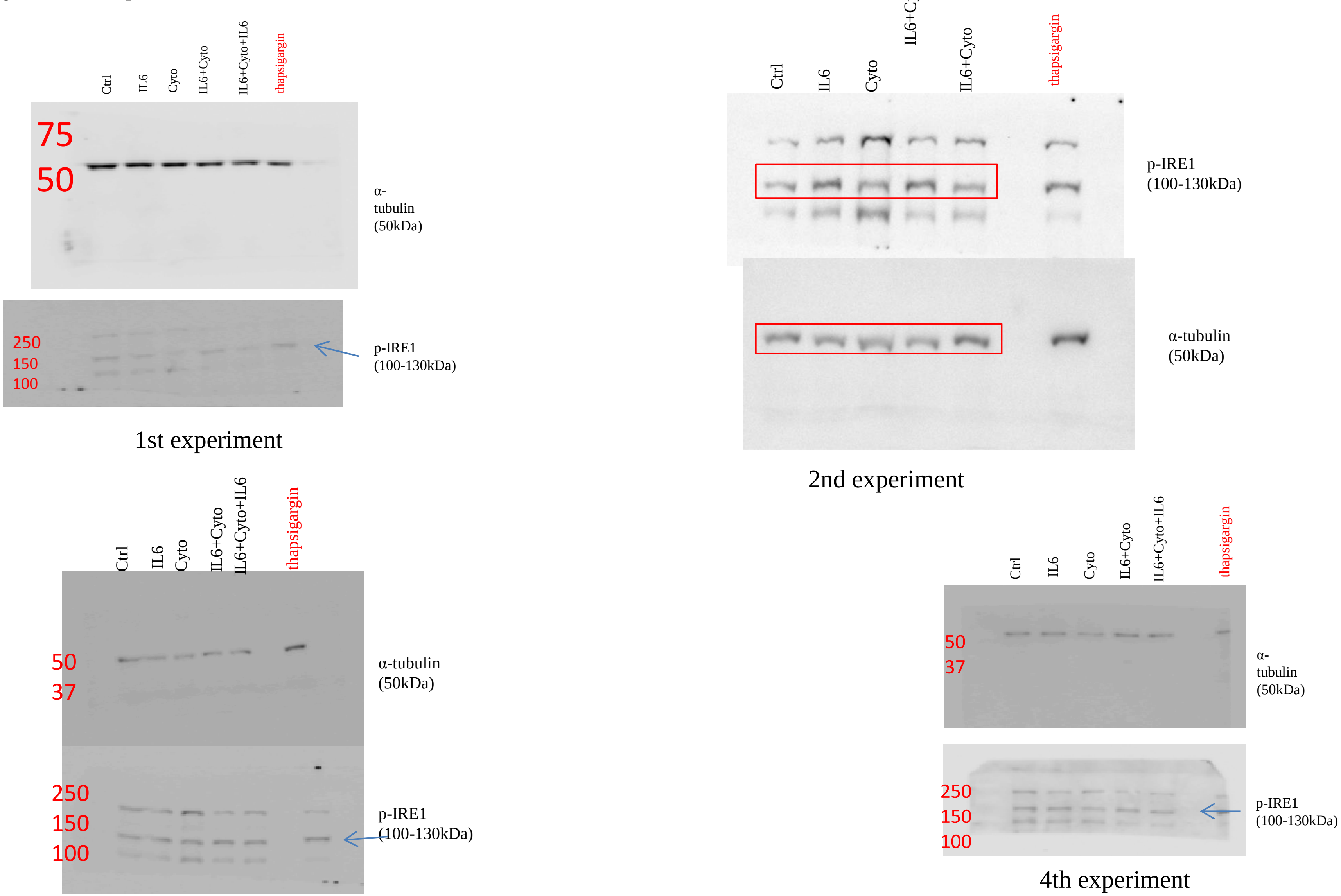

Figure 4 supplementary: Blots for p-IRE1a and a-tubulin in INS-1E cells. All 4 independent experiments performed for evaluation of p-IRE1a expression (showed bellow for each experiment) showed in Figure 2 C-D (manuscript) and the correspondent a-tubulin (showed above in each experiment). The red rectangle evidentiate the part of the blot used in figure 2 (manuscript). Cells were exposed to IL-6 (IL6) or untreated (Ctrl). After 24h cells were exposed to IL-1β and IFN-γ in the absence (Cyto) or in the presence of pre-treatment with IL-6 (IL6+Cyto) or continuous treatment of IL-6 (IL6+IL6+Cyto).

Figure 3 A-B. Caspase-3/α-tubulin

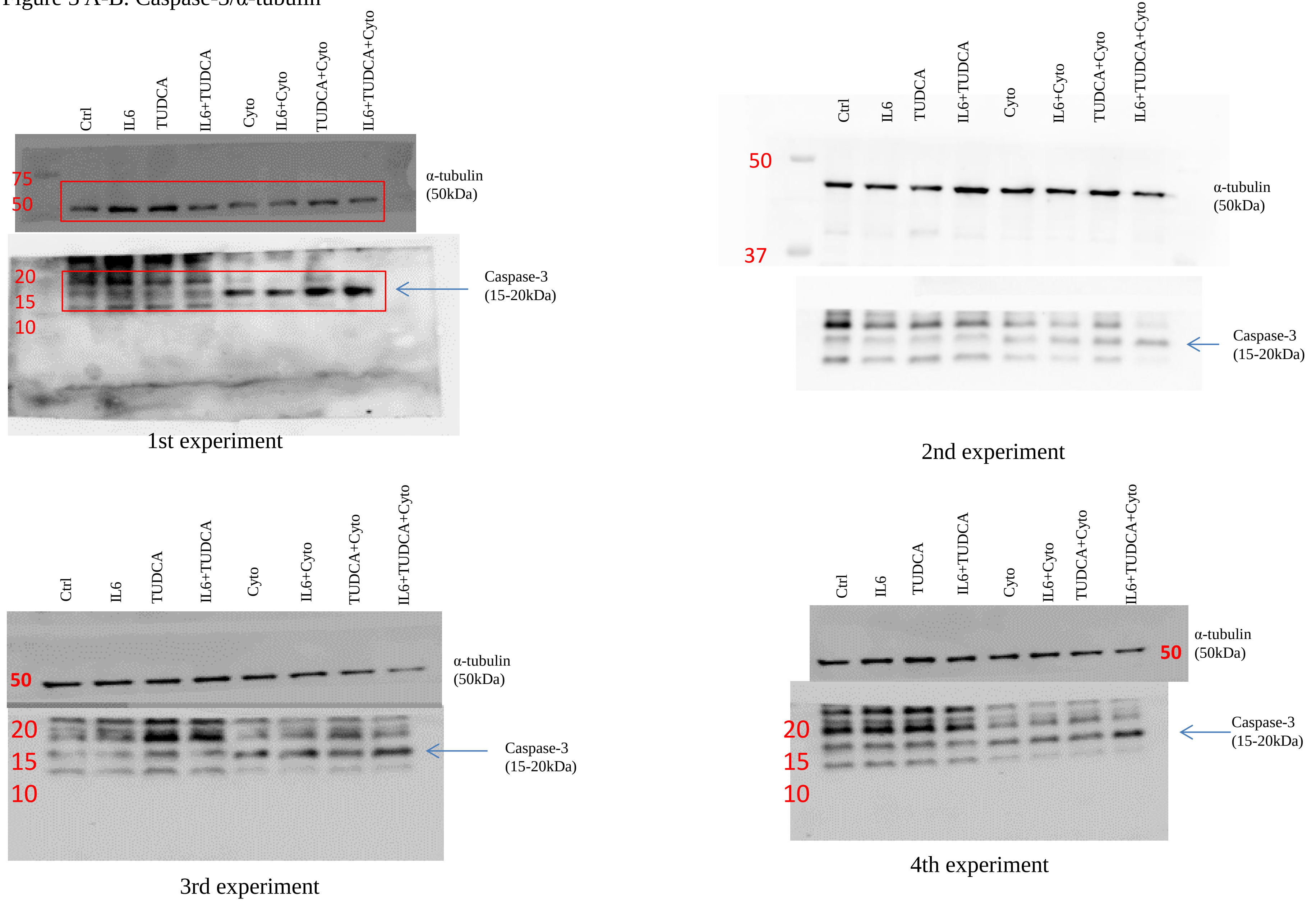

Figure 3 A-B. Caspase-3/α-tubulin

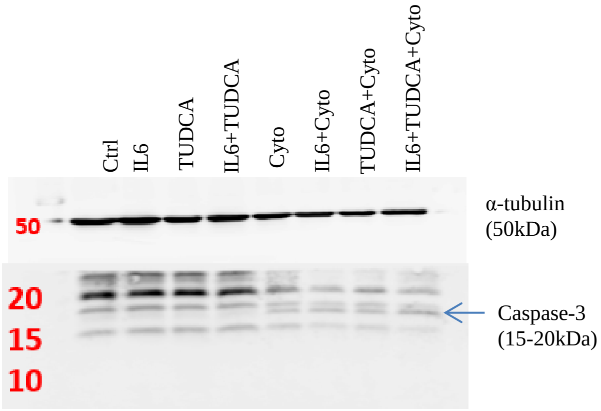

5th experiment

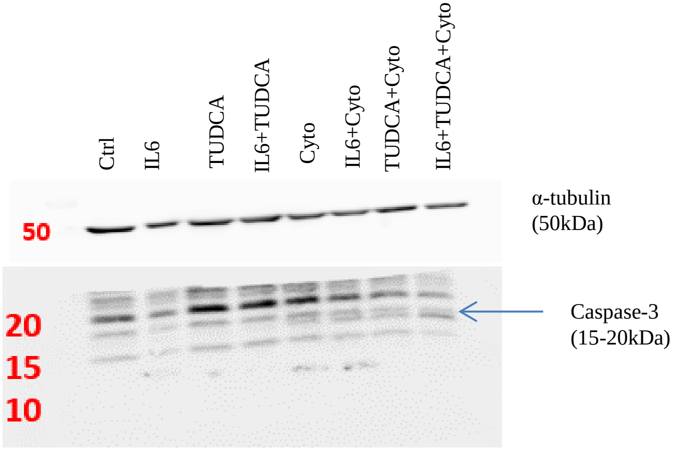

6th experiment

Figure 5 supplementary: Blots for Caspase 3 and a-tubulin in INS-1E cells. All 6 independent experiments performed for evaluation of Caspase 3 expression (showed bellow for each experiment) showed in Figure 3 A-B (manuscript) and the correspondent a-tubulin (showed above in each experiment). The red rectangle evidentiate the part of the blot used in figure 3 (manuscript). Cells were exposed to IL-6 (IL6) or untreated (Ctrl). After 24h cells were exposed to TUDCA in the absence (TUDCA) or in the presence of pre-treatment with IL-6 (IL6+TUDCA) and IL-1β and IFN-γ (TUDCA+Cyto) or pre-treatment with IL-6 and IL-1β and IFN-γ (IL6+TUDCA+Cyto). Cells also were exposed to IL-1β and IFN-γ in the absence (Cyto) or in the presence of pre-treatment with IL-6 (IL6+Cyto)).

Figure 3 C-D. p-eIF2α/α-tubulin

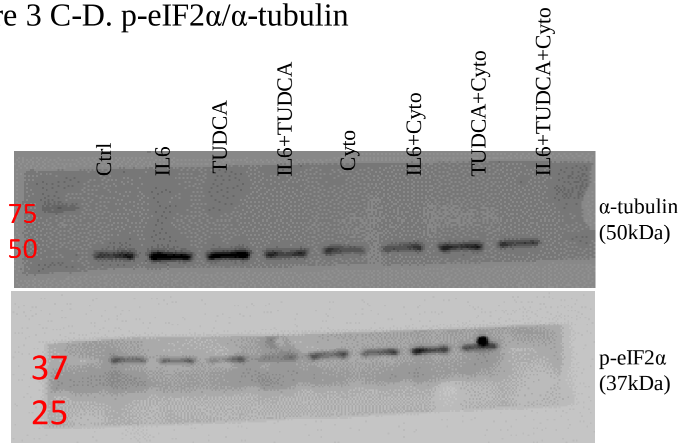

1st experiment

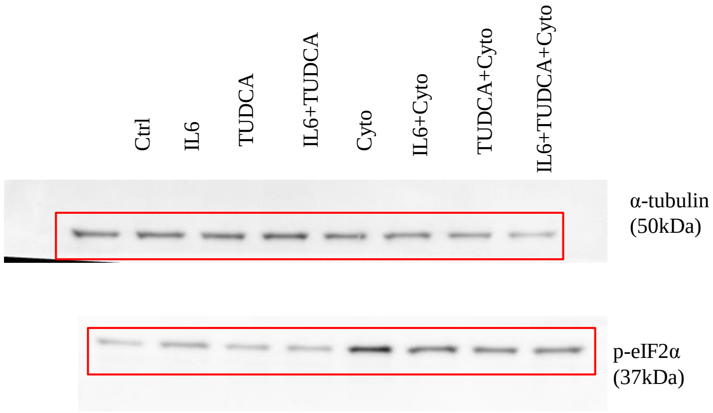

2nd experiment

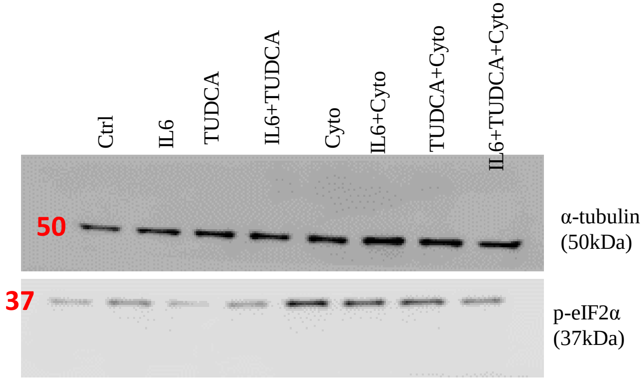

3rd experiment

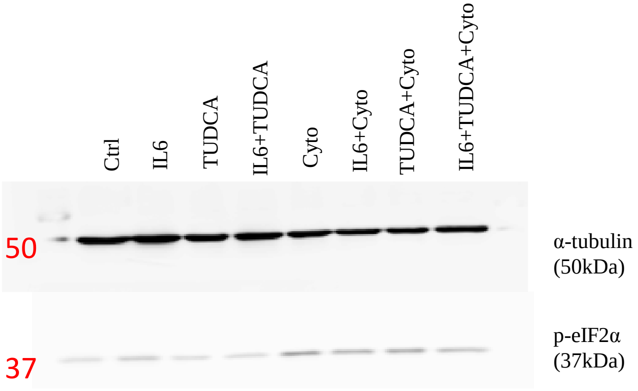

4th experiment

Figure 6 supplementary: Blots for eIF2a and a-tubulin in INS-1E cells. All 5 independent experiments performed for evaluation of eIF2a expression (showed bellow for each experiment) showed in Figure 3 C-D (manuscript) and the correspondent a-tubulin (showed above in each experiment). The red rectangle evidentiate the part of the blot used in figure 3 (manuscript). Cells were exposed to IL-6 (IL6) or untreated (Ctrl). After 24h cells were exposed to TUDCA in the absence (TUDCA) or in the presence of pre-treatment with IL-6 (IL6+TUDCA) and IL-1β and IFN-γ (TUDCA+Cyto) or pre-treatment with IL-6 and IL-1β and IFN-γ (IL6+TUDCA+Cyto). Cells also were exposed to IL-1β and IFN-γ in the absence (Cyto) or in the presence of pre-treatment with IL-6 (IL6+Cyto)).

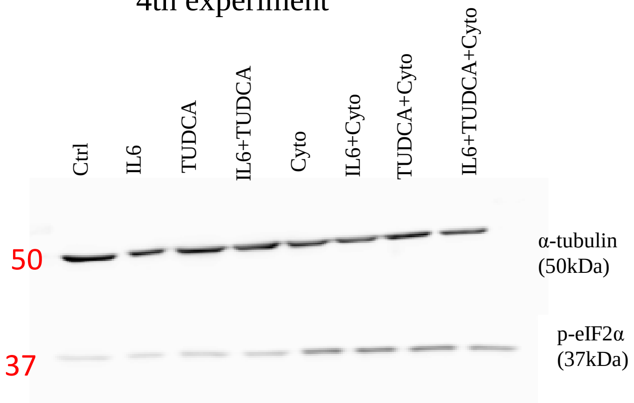

5th experiment
